# ASA-score is associated with 90-day mortality after complicated mild traumatic brain injury – a retrospective cohort study

**DOI:** 10.1007/s00701-024-06247-z

**Published:** 2024-09-11

**Authors:** Olivia Kiwanuka, Philipp Lassarén, Anders Hånell, Lennart Boström, Eric P. Thelin

**Affiliations:** 1 Department of Clinical Neuroscience, Karolinska Institutet, Stockholm, Sweden; 2 Department of Clinical Science and Education, Karolinska Institutet, Södersjukhuset, Stockholm, Sweden; 3 Department of Medical Sciences, Neurosurgery, Uppsala University, Uppsala, Sweden; 4 Department of Neurology, Karolinska University Hospital, Stockholm, Sweden

**Keywords:** Mild traumatic brain injury, Co-morbidity, Mortality, Prediction, Elderly, ASA score, Complicated mTBI

## Abstract

**Purpose:**

This study explores the association of the American Society of Anesthesiologists (ASA) score with 90-day mortality in complicated mild traumatic brain injury (mTBI) patients, and in trauma patients without a TBI.

**Methods:**

This retrospective study was conducted using a cohort of trauma patients treated at a level III trauma center in Stockholm, Sweden from January to December 2019. The primary endpoint was 90-day mortality. The population was identified using the Swedish Trauma registry. The Trauma and Injury Severity Score (TRISS) was used to estimate the likelihood of survival. Trauma patients without TBI (NTBI) were used for comparison. Data analysis was conducted using R software, and statistical analysis included univariate and multivariate logistic regression.

**Results:**

A total of 244 TBI patients and 579 NTBI patients were included, with a 90-day mortality of 8.2% (n = 20) and 5.4% (n = 21), respectively. Deceased patients in both cohorts were generally older, with greater comorbidities and higher injury severity. Complicated mTBI constituted 97.5% of the TBI group. Age and an ASA score of 3 or higher were independently associated with increased mortality risk in the TBI group, with odds ratios of 1.04 (95% 1.00–1.09) and 3.44 (95% CI 1.10–13.41), respectively. Among NTBI patients, only age remained a significant mortality predictor. TRISS demonstrated limited predictive utility across both cohorts, yet a significant discrepancy was observed between the outcome groups within the NTBI cohort.

**Conclusion:**

This retrospective cohort study highlights a significant association between ASA score and 90-day mortality in elderly patients with complicated mTBI, something that could not be observed in comparative NTBI cohort. These findings suggest the benefit of incorporating ASA score into prognostic models to enhance the accuracy of outcome prediction models in these populations, though further research is warranted.

**Supplementary Information:**

The online version contains supplementary material available at 10.1007/s00701-024-06247-z.

## Introduction

Mild Traumatic Brain Injury (mTBI) has emerged as a substantial burden on global health systems as it constitutes 60–95% of the 50–60 million TBI cases each year [9, 23]. Traditionally, mTBI is internationally defined as an admission Glasgow Coma Scale (GCS) of 13–15 [44], where some use the term “complicated” mild TBI in cases with radiologically visible intracranial lesions [23]. Outcomes are considered to be generally favorable, and current mild TBI-specific prediction models, such as Corticosteroid Randomization After Significant Head Injury (CRASH) [6], NIMJEN-Rubics [16], UPFRONT [45], and Toronto Rehabilitation Institute Concussion Outcome Determination and Rehab Recommendations (TRICORDRR) score [22], focus on either complete recovery (a Glasgow Outcome Score (GOS) of 5, or an extended GOS of 8) or lingering post-concussion symptoms [26]. Apart from psychiatric conditions, none of these models take pre-injury comorbidities into account and focus solely on trauma specific severity assessments.

As the global population ages, particularly in high-income countries, there's a notable increase in the incidence of mTBI among the elderly, primarily due to falls and other low-energy accidents [13, 23]. Studies have consistently shown that elderly individuals are more susceptible to adverse outcomes following mTBI, such as unfavorable functional outcome and death [4, 30, 37, 39, 41], where frailty and existing comorbidities are believed to be a contributing factor [13, 42, 49]. A common clinical tool used when assessing comorbidities is the American Society of Anesthesiologists (ASA) score. The ASA score is primarily used to assess patients preoperatively, and even though it is hampered some due to interrater variability it is still considered an important tool for risk assessment [25]. Studies indicate that ASA score might be an independent predictor of mortality after trauma [21, 40], however the association is poorly studied in TBI in general and mTBI in particular.

The initial management of mTBI does not usually involve neurosurgical intervention and is primarily handled by emergency physicians or general surgeons. Neurosurgeons are, however, often consulted if there are intracranial lesions, where they are tasked to provide advice in terms of management and outcome prediction of these patients. The Trauma and Injury Severity Score (TRISS), a general trauma prediction model of mortality, has been applied with some success to TBI [29, 47], but its efficacy in mTBI remains untested. Trauma patients without TBI therefore become an intriguing control group for studying the impact of mTBI on patient outcomes.

We previously conducted a prospective follow-up study of a mTBI cohort, where the main outcome metrics where depression and long-term health-related quality of life (HRQoL) [20]. We noted a strong correlation of pre-injury ASA score to outcome, but also a surprisingly high mortality rate in the, per GCS definitions, mTBI cohort, something seen in other studies as well [4, 41]. Mortality is not considered an expected outcome after mTBI which prompted us to explore this further and evaluate if the pre-trauma health status measured with ASA score can predict 90-day mortality post mTBI.

## Methods

### Study design

The study is a retrospective, single-center cohort study based on a cohort treated for trauma at Södersjukhuset, Stockholm, Sweden, January – December 2019. The hospital is one of several level III trauma centers in Stockholm, serving approximately 700 000 inhabitants. Stockholm also has a level I trauma center with neurosurgical capabilities, and patients with moderate-severe TBI (GCS 3–12) or polytrauma are typically referred there. Relevant baseline data prospectively assembled at the time of admission was retrospectively screened. The research protocol was approved by the Swedish Ethical Review Authority (Dnr: 2019–06122). The primary endpoint is mortality within the first 90-days from trauma.

### Population

The Swedish Trauma registry (SweTrau) was used to identify the trauma population. SweTrau, a national trauma registry established in 2011, is based on the revised Utstein Trauma Template [2]. Inclusion criteria for the current study was age ≥ 18 years at the time of injury, a trauma alert activated at hospital or a New Injury Severity Score (NISS) of more than 15, and GCS of 13 or higher. Exclusion criteria were trauma alert activated without underlying trauma, non-traumatic injuries such as asphyxia and hypothermia, death prior to arrival, trauma more than 24 h prior of admission, or missing significant data. In the case of multiple admissions during the set timeframe, only the first was included.

### Variables

Demographic data was collected from medical records. GCS at admission was assessed by the attending clinician upon hospital admission. Scoring of Abbreviated Injury Score (AIS) was performed by accredited AIS-scoring professionals according to the stated guidelines. GOS was obtained at discharge from the hospital. Anticoagulants were categorized as low-weight molecular heparin (LMH), direct oral anticoagulants (DOAK), or Warfarin. Antiplatelet medication included Clopidogrel and acetylsalicylic acid.

### ASA score

The ASA score has been shown to be a good approximation of comorbidities in trauma patients [33], and was in this study used to categorize illness before injury, according to the international ASA-PS edition, presented by the American Society of Anesthesiologist (supplementary Table 1) [7]. Severe comorbidity was defined as ASA-score ≥ 3.

### TBI vs NTBI

The cohort was divided into TBI, defined with the International Classification of Disease, 10th version (ICD-10), with all intracranial lesions and skull fractures classified as TBI (supplementary Table 2), and non-TBI (NTBI) subsequently.

### Trauma and Injury Severity Score, TRISS

TRISS is a widely recognized method used to predict the likelihood of survival following a traumatic injury. It combines the Revised Trauma Score (RTS), which includes physiological parameters like respiratory rate, systolic blood pressure, and GCS, with the Injury Severity Score (ISS) and the patient's age [1, 3]. The complete formula is outlined in supplementary Table 3. The result is a statistical model that estimates the probability of survival given the severity and nature of the injuries. This tool is particularly valuable in trauma research and clinical settings for assessing the effectiveness of care and for benchmarking outcomes.

### Statistical analysis

All data was analyzed using R [43] through the visual interface R-studio (v. 2022.07.2 Build 576, PBC, USA). Normal distribution was assessed with Shapiro–Wilk test. The results are presented as median with interquartile range for continuous data, and n (%) for nominal data, if not stated otherwise. Baseline characteristics among TBI and NTBI patients were assessed using the Mann–Whitney U test for quantitative variables, chi-squared test for categorical variables with expected count of at least 5, and Fischer’s exact test for categorical variables with expected count of less than 5, and ordered logit for ordinal variables. The significance level was set to 0.05. Univariable logistic regression was used to determine the effect of age, ASA score, and AIS (head) on mortality, and multivariable logistic regression was used to assess independency of ASA score and age.

## Results

### Demographics

**Fig. 1:**
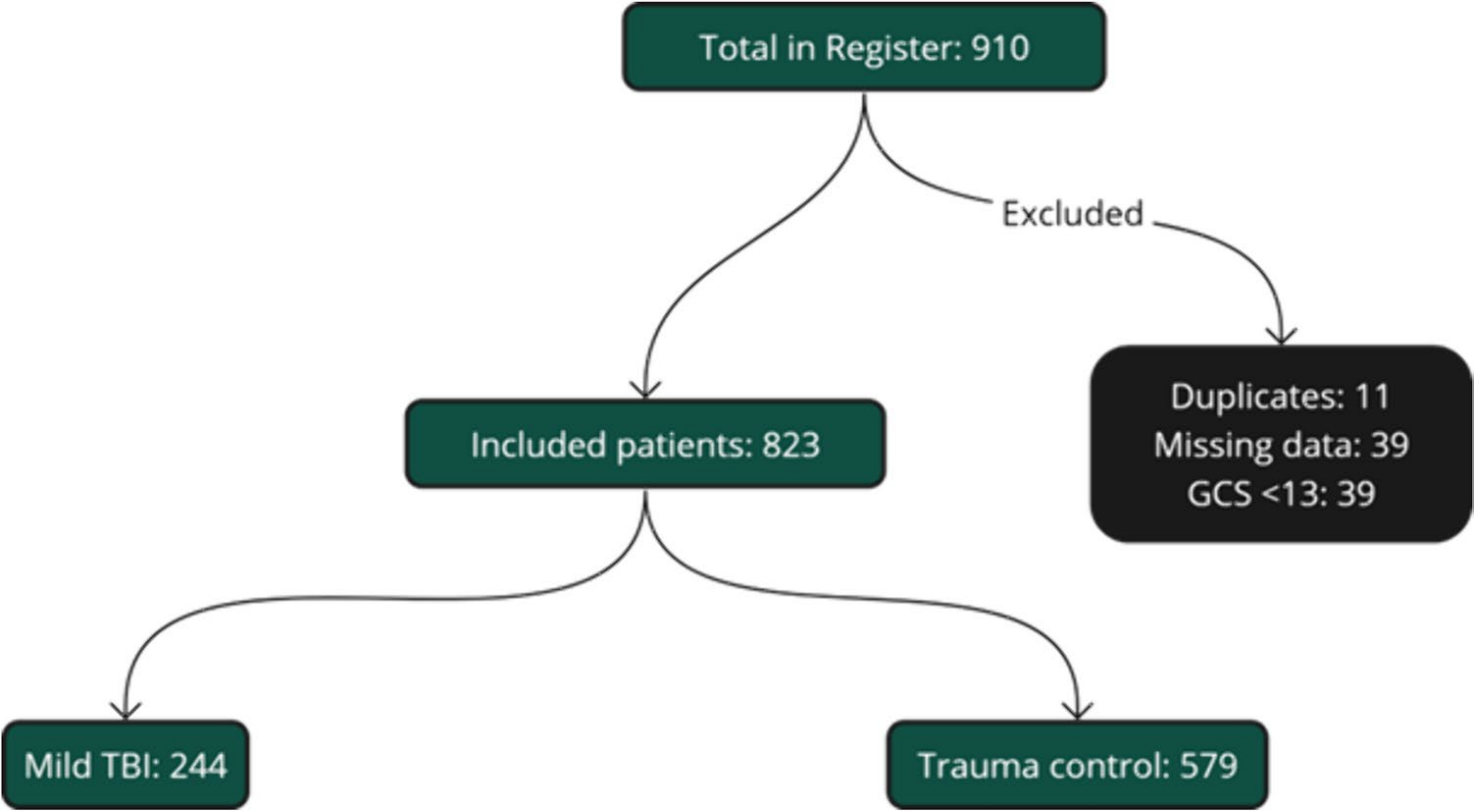
Inclusion flow chart for study participants. The chart summarizes the selection process from the initial registry containing 910 patients to the final inclusion cohort. Patients were excluded based on duplicates (n = 11), missing data (n = 39), and a Glasgow Coma Scale (GCS) score of less than 13 (n = 39), yielding 823 eligible participants. These were divided into two groups for analysis: mild TBI patients (n = 244) and trauma control patients (n = 579)

The study included 823 patients, with 244 in the TBI cohort and 579 in the NTBI cohort (Fig. 1). Gender distribution was similar, with 61% males in the TBI group and 57% in the NTBI group (p = 0.4) (Table 1). However, the median age was significantly higher in the TBI cohort (67 years) compared to the NTBI cohort (57 years) (p < 0.001). In terms of ASA scores, 39.4% of the TBI patients had an ASA score of 3 or higher, indicating severe systemic disease, while this was true for only 27% of the NTBI patients. Additionally, 41% of the NTBI cohort was classified as healthy (ASA 1), compared to 32% in the TBI cohort (p = 0.002). No significant difference was found in anticoagulant treatment, 66% of TBI patients and 77% of NTBI patients were not on any anticoagulants (p = 0.052).

**Table 1:** Demography of included patients with complete data. Results expressed in median and (IQR) as well as numeric values and (%). **Bold** is statistically significant. **ASA:** American Society of Anesthesiologists Classification, **GCS:** Glasgow Coma Scale, **NISS:** New Injury Severity Score, **AIS:** Abbreviated Injury Scale, **LMWH:**  low-molecular-weight heparin, **DOAK: **direct oral anticoagulants, **GOS:** Glasgow Outcome Score. Numerical median [IQR]

	**TBI**	**NTBI**	**p-value**
**Number**	244	579	
**Gender, Male (%)**	148 (61)	332 (57)	0.4
**Age**	67 [51, 78]	57 [37, 76]	** < 0.001**
**ASA (%)**			**0.002**
*1. Healthy*	78 (32)	239 (41)	
*2. Mild systemic disease*	69 (28)	183 (32)	
*3. Severe systemic disease*	96 (39)	152 (26)	
*4. Severe systemic disease constant threat to life*	1 (0.4)	5 (1)	
**Anticoagulants and antiplatelets (%)**			0.052
None	162 (66)	448 (77)	
*Anticoagulants*			
LMH	1 (0.4)	4 (1)	
DOAK	24 (10)	38 (7)	
Warfarin	9 (4)	16 (3)	
*Antiplatelets*			
Clopidogrel	9 (4)	18 (3)	
Acetylsalicylic acid	32 (13)	46 (8)	
**Mechanism of injury**			** < 0.001**
*Traffic -car*	1 (0.4)	109 (19)	
*Traffic -motorcycle*	4 (1.6)	15 (3)	
*Traffic- bicycle*	30 (12)	37 (6)	
*Traffic pedestrian*	5 (2)	12 (2)	
*Traffic other*	0 (0)	2 (0.3)	
*Low energy fall*	143 (59)	219 (38)	
*High energy fall*	41 (17)	107 (19)	
*Blunt object*	20 (8)	42 (7)	
*Firearm*	0 (0)	1 (0.2)	
*Stabbing*	0 (0)	33 (6)	
**NISS**	14 [9, 21]	3 [2, 11]	** < 0.001**
**TRISS**	1.00 [0.99, 1.00]	1.00 [0.99, 1.00]	** < 0.001**
**MT** = no (%)	0 (0)	2 (6)	0.096
**GCS (%)**			** < 0.001**
13	8 (3)	8 (1)	
14	76 (31)	55 (10)	
15	160 (66)	516 (89)	
**AIS (head) (%)**			** < 0.001**
0	1 (0.4)	431 (74)	
1	5 (2)	143 (25)	
2	97 (40)	2 (0.3)	
3	93 (38)	3 (0.5)	
4	31 (13)	0 (0)	
5	17 (7)	0 (0)	
**Stay (median [IQR])**	3 [2, 5]	2 [1, 4]	** < 0.001**
**GOS (%)**			** < 0.001**
1	7 (3)	8 (1)	
3	69 (28)	145 (25)	
4	166 (68)	199 (34)	
5	2 (1)	227 (39)	
**90d mortality (%)**	20 (8.2)	31 (5.4)	0.2

### Injury characteristics

The mechanism of injury varied significantly between the cohorts (p < 0.001) (Table 1). Low energy falls were the most common mechanism in both groups but were more prevalent in the TBI cohort (59%) compared to the NTBI cohort (38%). Traffic-related injuries were more common in the NTBI group (30%) than in the TBI group (16%).

**Fig. 2:**
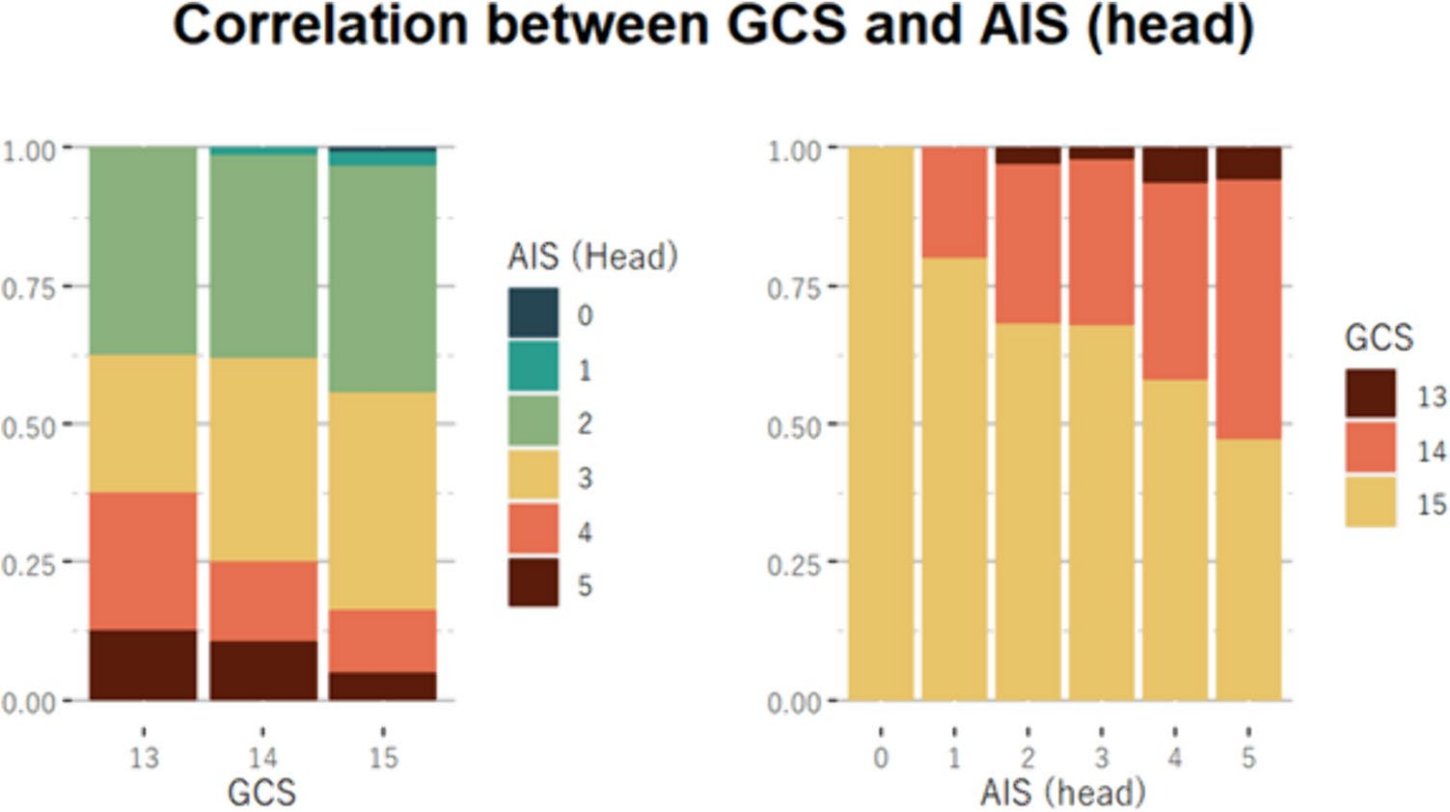
Correlation between Glasgow Coma Scale (GCS) Scores and Abbreviated Injury Score (AIS) for Head Injuries in TBI Patients. For the TBI cohort, the left panel displays the AIS distribution for varying GCS categories, elucidating the severity of head injuries alongside consciousness levels. The right panel depicts the GCS scores within AIS categories, illustrating the impact of head injury severity at initial assessment. Notably, 97.5% of these patients were classified with 'mild complicated TBI,' characterized by intracranial lesions but maintaining GCS scores of 13 to 15, indicating less severe impairment of consciousness despite significant imaging findings. Each color corresponds to a different AIS score from 0 (indicating no injury) to 5 (indicating critical injury)

The TBI cohort had significantly higher injury severity as indicated by NISS (14 vs. 3, p < 0.001) and a slightly higher TRISS. The GCS score was lower in the TBI cohort (p < 0.001), with 66% having a GCS of 15, compared to 89% in the NTBI cohort. Additionally, 97,6% of the TBI patients had a complicated mTBI (AIS head score ≥ 2) (Fig. 2).

### Outcome

The median length of hospital stay was longer for TBI patients at 3 days, compared to 2 days for NTBI patients (p < 0.001) (Table 1). The GOS scores indicated worse outcomes for the TBI cohort, with only 1% achieving a GOS of 5 compared to 39% of NTBI patients (p < 0.001). The 90-day mortality rate was 8.2% (20 patients) for the TBI group and 5.4% (31 patients) for the NTBI group (p = 0.2). Deceased NTBI patients were significantly older than deceased TBI patients (86 vs. 77 years, p = 0.043) (Table 2). While ASA scores were similar (p = 0.5), NTBI deceased patients had lower injury severity (NISS 10 vs. 21, p < 0.001) and a lower TRISS (0.993 vs. 0.996, p = 0.001). Mortality timing showed that 55% of deaths in both cohorts occurred within 90 days. In-hospital mortality was 35% for TBI patients and 26% for NTBI patients (p = 0.6).

**Table 2:** Deceased patients in the TBI and NTBI cohorts. Results expressed in median and (IQR) as well as numeric values and (%). **Bold** is statistically significant. **ASA:** American Society of Anesthesiologists Classification, **GCS:** Glasgow Coma Scale, **NISS:** New Injury Severity Score, **AIS:** Abbreviated Injury Scale, **LMWH: **low-molecular-weight heparin, **DOAK: ** direct oral anticoagulants, **GOS:** Glasgow Outcome Score. Numerical median [IQR]

	**TBI**	**NTBI**	**p-value**
**Number**	20	31	
**Gender, Male (%)**	13 (65)	14 (45)	0.3
**Age**	77 [73, 83]	86 [77, 93]	**0.043**
**ASA (%)**			0.5
*1. Healthy*	0 (0)	0 (0)	
*2. Mild systemic disease*	4 (20)	10 (32)	
*3. Severe systemic disease*	15 (75)	18 (58)	
*4. Severe systemic disease constant threat to life*	1 (5)	3 (10)	
**Anticoagulants and antiplatelets (%)**			0.4
None	7 (47)	11 (41)	
*Anticoagulants*			
LMH	0 (0)	2 (7)	
DOAK	6 (40)	5 (19)	
Warfarin	0 (0)	2 (7)	
*Antiplatelets*			
Clopidogrel	0 (0)	1 (4)	
Acetylsalicylic acid	2 (13)	6 (22)	
**Mechanism of injury**			0.5
*Traffic -car*	0 (0)	2 (6)	
*Traffic -motorcycle*	0 (0)	0 (0)	
*Traffic- bicycle*	0 (0)	0 (0)	
*Traffic pedestrian*	0 (0)	0 (0)	
*Stabbing*	0 (0)	0 (0)	
*Blunt object*	0 (0)	1 (3)	
*Low energy fall*	18 (90)	26 (84)	
*High energy fall*	2 (10)	2 (6)	
*Car-Motorcycle*	0 (0)	0 (0)	
*Firearm*	0 (0)	0 (0)	
*Stab*	0 (0)	0 (0)	
*Traffic other*	0 (0)	0 (0)	
**NISS**	21 [12, 28]	10 [5, 14]	** < 0.001**
**TRISS**	0.996 [0.993, 0.997]	0.993 [0.990, 0.993]	**0.001**
**MT** = no (%)	0 (0)	2 (6)	0.096
**GCS (%)**			**0.011**
13	4 (20)	0 (0.0)	
14	9 (45)	10 (32)	
15	7 (35)	21 (68)	
**AIS (head) (%)**			> 0.9
0	0 (0)	23 (74)	
1	0 (0)	8 (26)	
2	4 (20)	0 (0)	
3	7 (35)	0 (0)	
4	6 (30)	0 (0)	
5	3 (15)	0 (0)	
**Stay (median [IQR])**	6 [4, 12]	5 [3, 11]	0.5
**Mortality (%)**			0.6
In-hospital	7 (35)	8 (26)	
30 day	2 (10)	6 (19)	
90 day	11 (55)	17 (55)	

**Fig. 3:**
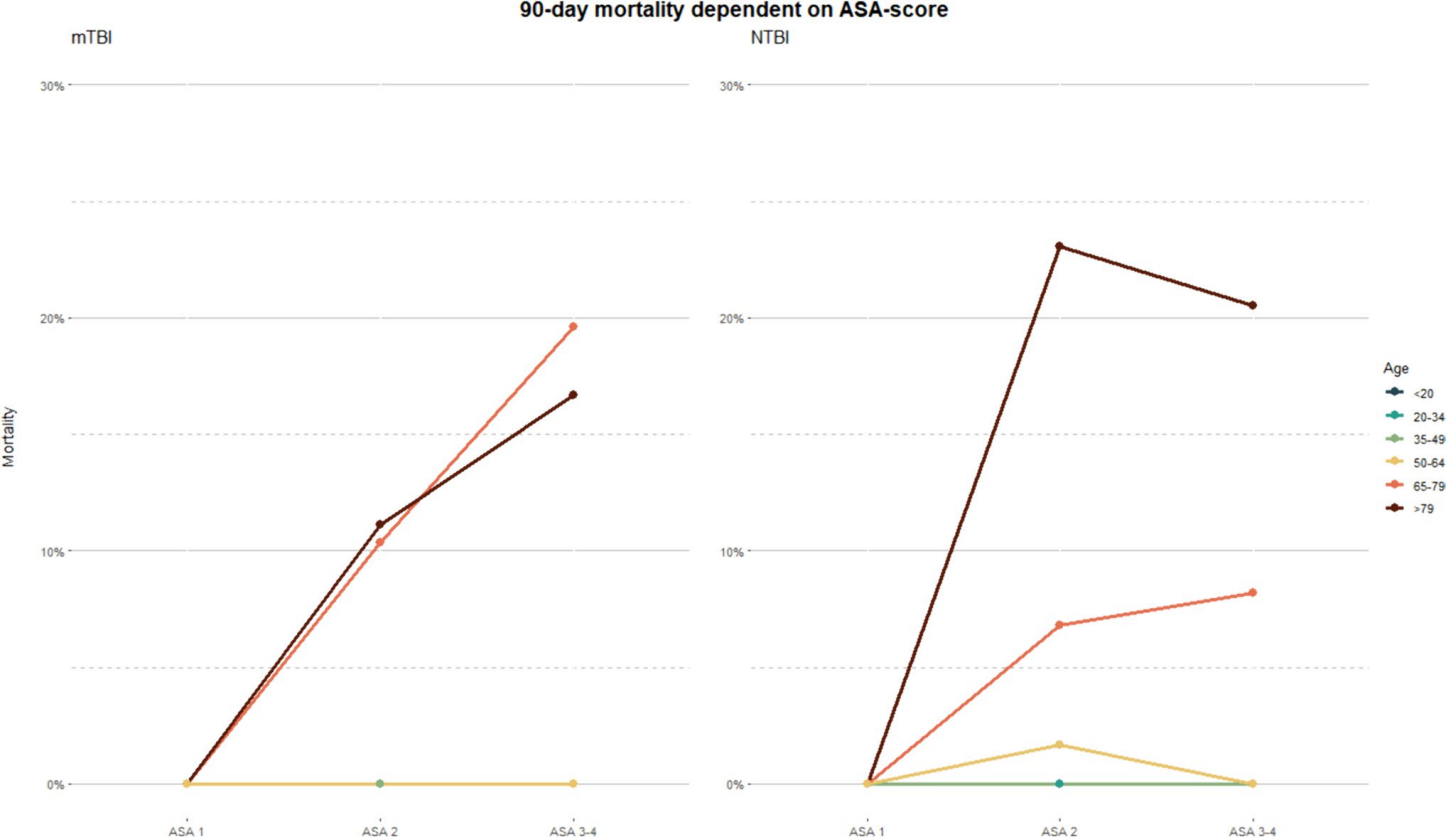
90-Day Mortality Rates by ASA-Score in mTBI and Trauma Patients. Mortality rates are segmented by ASA categories 1, 2, and ≥ 3. Notably, there is a marked increase in mortality rates with higher ASA scores, especially in patients aged ≥ 65. The mTBI graph shows a steep rise in mortality rates correlated with an ASA score of ≥ 3, with an odds ratio (OR) for mortality at 5.05, signifying a substantial risk increase. In contrast, the trauma graph illustrates a less pronounced increase in mortality with an ASA score of ≥ 3, with an OR of 0.7, which did not reach statistical significance. This trend reflects the study's results where the ASA score was a strong independent predictor of mortality in the TBI group

In the univariable analysis age, an ASA score, or an AIS (head) of 3 or higher, and treatment with anticoagulants were associated with 90-day mortality in the TBI group (Table 3 and Fig. 3). Both age and ASA maintained an independent association for mortality in the multivariable analysis, with odds ratios of 1.04 (1.00–1.09) and 3.44 (1.10–13.41), respectively.

**Table 3:** Univariable and multivariable logistic regression analyses of mortality. The association of selected parameters to 90-day mortality. Age, ASA score, AIS (head), and the use of anticoagulants, were associated with mortality in the univariable analysis for the TBI-population. Age and ASA score remained significant in the multivariable analysis. Age, ASA score, anticoagulants, and antiplatelets were associated with mortality in the univariable analysis for the NTBI-population, but only age was significant in the multivariable analysis. **Bold** is statistically significant. **ASA:** American Society of Anesthesiologists Classification, **GCS:** Glasgow Coma Scale, **NISS:** New Injury Severity Score, **AIS:** Abbreviated Injury Scale

TBI						
	**Univariable**			**Multivariable**		
	**OR**	**95% CI**	**p-value**	**OR**	**95% CI**	**p-value**
**Age**	1.06	1.03 to 1.10	**0.001**	1.04	1.00 to 1.09	**0.034**
**Sex**						
* Female*	—	—				
* Male*	1.22	0.48 to 3.37	0.7			
**Preinjury ASA**						
* 1 to 2*	—	—		—	—	
* 3 and above*	7.06	2.49 to 25.3	** < 0.001**	3.44	1.10 to 13.41	**0.048**
**AIS head**						
* 1 to 2*	—	—				
* 3 and above*	3.11	1.10 to 11.1	**0.048**			
**Anticoagulants and antiplatelets**						
* None*	—	—				
* Anticoagulants*	4.74	1.43 to 15.3	**0.009**			
* Antiplatelets*	1.14	0.16 to 4.92	0.9			
**NTBI**						
	**Univariable**			**Multivariable**		
	**OR**	**95% CI**	**p-value**	**OR**	**95% CI**	**p-value**
**Age**	1.10	1.07 to 1.14	** < 0.001**	1.10	1.06 to 1.14	** < 0.001**
**Sex**						
* Female*	—	—				
* Male*	0.60	0.28 to 1.23	0.2			
**Preinjury ASA**						
* 1 to 2*	—	—		—	—	
* 3 and above*	6.36	2.99 to 14.4	** < 0.001**	1.19	0.51 to 2.94	**0.6**
**AIS head**						
* 1 to 2*	—	—				
* 3 and above*	0.96	0.44 to 2.33	0.9			
**Anticoagulants and antiplatelets**						
* None*	—	—				
* Anticoagulants*	7.30	2.82 to 18.5	** < 0.001**			
* Antiplatelets*	4.88	1.73 to 12.9	**0.002**

Age, ASA score, and both anticoagulants and antiplatelets were associated with 90-day mortality in the univariable analysis for the NTBI-group, however for the NTBI patients, ASA was not statistically significant in the multivariable analysis (Table 3 and Fig. 3).

TRISS calculations revealed a small but statistically significant difference in predicted survival between the TBI and NTBI groups overall (p < 0.001), as well as within the deceased cohorts (0.996 [95% CI: 0.993–0.997] for TBI vs. 0.993 [95% CI: 0.990–0.993] for NTBI, p < 0.001) (Table 1 and Table 2).

## Discussion

This retrospective cohort study highlights a significant association between ASA score and 90-day mortality in patients with complicated mTBI, that is not found in the NTBI cohort.

Mild TBI is typically not associated with high mortality rates. However, our study found a 90-day mortality rate of 8.2% for mTBI patients, compared to 5.4% for NTBI trauma patients, exclusively among the elderly. The high mortality reflects the significant impact of demographic shifts, indicating that complicated mTBI in older adults can no longer be considered "mild." This is illustrated in a 2024 study Orso et al. [31], which reported a seven percent 90-day mortality rate in mTBI patients, with no cases younger than 80 years. This mortality rate is comparable to that seen in elderly hip fracture patients, which ranges from 7.8% to 9.4% [38]. The demographic shift necessitates a re-evaluation of mTBI management, as older adults tend to sustain more severe injuries despite having a similar admission GCS score [41]. Our data supports this, showing that over 97% of mTBI patients had an AIS head score of 2 or more, a level some argue should classify as moderate to severe TBI [36]. Additionally, 68% of our cohort had isolated mTBI, defined by an AIS score below 2 in other body regions.

Although we lack specific data on the causes of death due to limitations of the SweTrau registry, insights can be drawn from similar conditions. Mortality after hip fractures is often attributed to pulmonary infections, myocardial infarction, and sepsis, primarily due to underlying comorbidities and immobilization [18]. Given the similarities in demographics, these factors are likely contributors to mortality in mTBI patients as well. The extensive study by Dolejs and Marešová assessed age-related increases in mortality due to non-traumatic causes in Scandinavian countries, revealing a yearly mortality rate of about 0.05% for individuals aged 18–44, increasing to 0.5% for those aged 65–74, and up to 3% for those 85 and older [10]. Even when compared to the oldest age group, our 90-day mortality rates are significantly higher, highlighting the compounded impact of age and trauma on resilience and outcomes in elderly mTBI patients. This aligns with research indicating that elderly TBI patients have worse mortality and functional outcomes than younger patients with similar injury severity [41], and that pre-existing medical conditions increase mortality risk in elderly trauma patients, especially for minor to moderate injuries [5]. The severity of the TBI likely contributes to our high mortality rate as well, as an AIS head score of 3 or higher was associated with increased mortality, consistent with previous findings [35]. While the TBI itself may not directly cause death (e.g., through herniation or ischemia), its severity can impact factors such as mobilization, leading to indirect detrimental effects like aspiration or embolism.

Our main finding is that ASA score, adjusted for age, was independently associated with mortality. The overall burden of disease that the ASA score encompasses, captures both the impact of comorbidities [33] and functional frailty [25]. The literature on the role of comorbidities in TBI outcomes is conflicted. For instance, Orso et al. found no statistically significant correlation between mortality at 90 days and the presence of specific comorbidities (p-value 0.177) [31]. In a 2021 systematic review by Xiong et al. the authors noted that while the absolute number and presence of comorbidities were significantly associated with long-term mortality (> 1 year), they were not linked to short-term mortality [48]. Conversely, Dell et al. found in a study involving over 20,000 patients, that as the number of pre-existing health conditions increased, the mortality rate after TBI climbed incrementally by a factor of 1.7–1.9, suggesting a clear relationship between comorbidity burden and mortality risk [8]. A recent meta-analysis by Roohollahi found that five out of nine studies investigating the association between frailty and in-hospital mortality after TBI reported a positive association [34]. However, the four studies that did not find frailty to be a significant predictor of in-hospital death had adjusted for age and other variables, complicating the conclusion. The association between ASA score and outcomes after TBI is not well-studied, despite its widespread use in clinical practice. However, its predictive value has been extensively demonstrated in surgical contexts. Hackett et al. reported that the ASA score independently predicts 30-day complication and mortality rates across a wide spectrum of surgical procedures [14]. Specifically, their study found a 30-day mortality rate of 1.41% for patients with an ASA score of 3, increasing to 11.14% for those with an ASA score of 4. Our study observed a 30-day mortality rate of 3.7% in TBI patients, predominantly those with ASA scores of 3 (ranging from 2 to 4), aligning with these findings. Additionally, we found a strong correlation between ASA score and 90-day mortality in mTBI patients (p < 0.001), independent of age (p = 0.048), indicating its potential as a valuable predictive marker for TBI outcomes.

We did not observe the same independent result of ASA score in our NTBI cohort, despite literature suggesting otherwise. Previous studies have demonstrated a strong association between pre-existing medical conditions and increased in-hospital mortality after trauma [17, 19, 40, 46]. In our study, the deceased NTBI cohort was older than the deceased TBI cohort (86 vs. 77 years, p = 0.043) but had similar ASA score (p = 0.462). NTBI patients were significantly less injured (NISS 10 vs. 22, p < 0.001) yet had a slightly lower TRISS (0.99 vs. 1, p < 0.001). Several possible aspects might explain this difference. Firstly, there might be a treatment bias and under-triaging of mTBI compared to NTBI patients. Research indicates treatment intensity for elderly TBI patients is reduced compared to younger adults, suggesting that a treatment bias [39], and under-triaging [24, 35], exist. Although treatment bias and under-triaging can occur in NTBI patients as well, the nature of treatment for mTBI differs significantly. In our setting, the absence of neurosurgical care compared to readily available trauma and orthopedic surgery means that the threshold for transferring an mTBI patient to a higher level of care is likely higher. This limitation can potentially impact outcomes more significantly in mTBI patients, as delays or omissions in specialized treatment might exacerbate their condition. Secondly, it might be due to inclusion bias as the SweTrau registry excludes isolated hip fractures and thereby the frailest NTBI patients. Thirdly, elderly patients who do not sustain a TBI after a trauma might demonstrate a higher resilience compared to those who do. This hypothesis is strengthened by the fact that TRISS showed a small, but statistically significant difference between the outcome of NTBI and not mTBI. TRISS calculates a survival probability based on anatomical and physiological data and is widely established in trauma research and to measure performance of trauma care systems [11, 32]. Studies have also shown its usefulness in severe TBI [29, 47], and its limited effectiveness in our study indicate that factors beyond the immediate scope of the injury, such as pre-existing health conditions captured by the ASA score, play a more crucial role in determining mortality in mTBI patients. Adding ASA score to TRISS can improve its accuracy in predicting mortality [12], and emphasizes the value of including pre-injury health information in models predicting outcomes, especially in less severe trauma cases.

The TBI cohort had worse GOS scores at discharge compared to NTBI, which likely reflect the older age and higher ASA scores in this group. This finding indicates a greater burden of pre-existing health conditions and may not fully capture the long-term functional outcomes. As GOS scores at discharge primarily reflect acute recovery, they might underestimate the potential for longer-term improvement, especially since no pre-injury GOS scores were available for comparison.

This study has several limitations that warrant consideration. Firstly, the study was conducted at a single level III trauma center, which provided a controlled environment for data collection and analysis but may limit the generalizability of our findings. However, these centers manage a majority of mTBI patients making it a relevant setting for this cohort. Secondly, the majority of the mTBI cohort had intracranial findings, compared to the usual 10% of mTBI patients [27], representing the more severe end of the mTBI spectrum. As the management of complicated mTBI is more complex, this group is highly relevant to study compared to mTBI patients without intracranial findings. Thirdly, the study has a relatively small sample size, with 244 TBI patients included and only 20 events for the primary outcome of 90-day mortality. The mortality rate is aligned with other studies of mTBI [4, 31, 41], and our exclusion rate due to missing data was low, suggesting that our cohort is relevant to typical level III trauma populations. Due to this, however, we have refrained from extensive multivariable analyses which would likely be underpowered and have instead focused on adjusting for only age and ASA, showing an independent association with mortality. Larger, multi-center studies will have to validate if ASA remains independent in presence of other relevant factors such as injury metrics (i.e. AIS). Finally, we selected 90-day mortality rather than 30-day mortality as our primary endpoint to capture the full scope of mortality attributable to TBI. Prior studies indicate that the risk of death may remain elevated beyond 30 days [15, 28, 50]. However, it is important to note that longer follow-up intervals increase the potential for confounding from trauma-unrelated causes of death. Despite this, we believe our findings offer significant insights into the factors affecting outcomes after mTBI. The ASA score, in particular, emerges as a relevant metric for prognostic models, providing additional predictive value beyond age and specific comorbid diagnoses.

## Conclusion

This retrospective cohort study highlights a significant association between ASA score and 90-day mortality in elderly patients with complicated mTBI, which is not observed in the NTBI cohort. These findings suggest the benefit of incorporating ASA score into prognostic models to enhance the accuracy of outcome predictions and guide treatment strategies in this vulnerable population.

## Supplementary Information

Below is the link to the electronic supplementary material.

Supplementary file1 (DOCX 15 KB)

Supplementary file2 (DOCX 15 KB)

Supplementary file3 (DOCX 17 KB)

Supplementary file4 (DOCX 16 KB)

## Data Availability

The data that support the findings of this study are available on request from the corresponding author OK. The data are not publicly available due to them containing information that could compromise research participant privacy.
