# Supplementary material for: ASA-score is associated with 90-day mortality after complicated mild traumatic brain injury – a retrospective cohort study
Source: Acta Neurochir (Wien). 2024 Sep 11;166(1):363. doi: 10.1007/s00701-024-06247-z (PMC11390782; doi:10.1007/s00701-024-06247-z)
Supplement: Supplementary file 1 — Supplementary file1 (DOCX 15 KB) [file 701_2024_6247_MOESM1_ESM.docx]

**Supplementary table 1**

| **ASA Score** | **Definition** |
| --- | --- |
| I | A normal healthy patient |
| II | A patient with mild systemic disease |
| III | A patient with severe systemic disease |
| IV | A patient with severe systemic disease that is a constant threat to life |
| V | A moribund patient who is not expected to survive without the operation |
| VI | A declared brain-dead patient whose organs are being removed for donor purposes |
