# Supplementary material for: ASA-score is associated with 90-day mortality after complicated mild traumatic brain injury – a retrospective cohort study
Source: Acta Neurochir (Wien). 2024 Sep 11;166(1):363. doi: 10.1007/s00701-024-06247-z (PMC11390782; doi:10.1007/s00701-024-06247-z)
Supplement: Supplementary file 2 — Supplementary file2 (DOCX 15 KB) [file 701_2024_6247_MOESM2_ESM.docx]

**Supplementary table 2**

| **Table 1. ICD-10 codes defining TBI** | |
| --- | --- |
| S02.0 | Fracture of vault of skull |
| S02.1 | Fracture of base of skull |
| S02.3 | Fracture of orbital floor |
| S02.7 | Multiple fractures involving skull and facial bones |
| S02.8 | Fractures of other skull and facial bones |
| S02.9 | Fracture of skull and facial bones, part unspecified |
| S06.0 | Concussion |
| S06.1 | Traumatic cerebral oedema |
| S06.2 | Diffuse brain injury |
| S06.3 | Focal brain injury |
| S06.4 | Epidural hemorrhage |
| S06.5 | Traumatic subdural hemorrhage |
| S06.6 | Traumatic subarachnoid hemorrhage |
| S06.7 | Intracranial injury with prolonged coma |
| S06.8 | Other intracranial injuries |
| S06.9 | Intracranial injury, unspecified |
