# Supplementary material for: ASA-score is associated with 90-day mortality after complicated mild traumatic brain injury – a retrospective cohort study
Source: Acta Neurochir (Wien). 2024 Sep 11;166(1):363. doi: 10.1007/s00701-024-06247-z (PMC11390782; doi:10.1007/s00701-024-06247-z)
Supplement: Supplementary file 3 — Supplementary file3 (DOCX 17 KB) [file 701_2024_6247_MOESM3_ESM.docx]

**Supplementary table 3**

| **Probability of survival (Ps)** | **P(s) = 1/ (1+ e^-b^)** | | |  |  |
| --- | --- | --- | --- | --- | --- |
| **b** | b = b_0_ + b_1_ x RTS + b_2_ x ISS + b_3_ x age index, | | | | |
|  | *blunt* | *penetrating* | |  |  |
| b0 | -0,4499 | -2,5355 |  |  |  |
| b1 | 0,8085 | 0,9934 |  |  |  |
| b2 | -0,0835 | -0,0651 |  |  |  |
| b3 | -1,743 | -1,136 |  |  |  |
| **Revised Trauma Score (RTS)** | RTS = 0,9386 (GCSc) + 0,7326 (SBPc) + 0,2908 (RRc) | | | | |
| *GCS* | *GCSc* |  |  |  |  |
| 13-15 | 4 |  |  |  |  |
| 9-12 | 3 |  |  |  |  |
| 6-8 | 2 |  |  |  |  |
| 4-5 | 1 |  |  |  |  |
| 3 | 0 |  |  |  |  |
| *SBP* | *SBPc* |  |  |  |  |
| >89 | 4 |  |  |  |  |
| 76-89 | 3 |  |  |  |  |
| 50-75 | 2 |  |  |  |  |
| 1-49 | 1 |  |  |  |  |
| 0 | 0 |  |  |  |  |
| *RR* | *RRc* |  |  |  |  |
| 10-29 | 4 |  |  |  |  |
| >29 | 3 |  |  |  |  |
| 6-9 | 2 |  |  |  |  |
| 1-5 | 1 |  |  |  |  |
| 0 | 0 |  |  |  |  |
| **ISS** | (1st AIS score)^2^+(2nd AIS score)^2^+(3rd AIS score)^2^ | | | | |
| **Age-index** |  | | | | |
| Age <55 | 0 | | | | |
| Age ≥55 | 1 | | | | |
