# Supplementary material for: ASA-score is associated with 90-day mortality after complicated mild traumatic brain injury – a retrospective cohort study
Source: Acta Neurochir (Wien). 2024 Sep 11;166(1):363. doi: 10.1007/s00701-024-06247-z (PMC11390782; doi:10.1007/s00701-024-06247-z)
Supplement: Supplementary file 4 — Supplementary file4 (DOCX 16 KB) [file 701_2024_6247_MOESM4_ESM.docx]

**Supplementary table 4**

|  | Alive | Deceased |
| --- | --- | --- |
| n (%) | 224 (91.8) | 20 (8.2) |
| Brain stem contusion | 1 (0.5) | 0 (0.0) |
| Brain stem hemorrhagic injury | 0 (0.0) | 1 (5.3) |
| Cerebellum contusion, small | 1 (0.5) | 0 (0.0) |
| Cerebral contusion, large | 2 (1.0) | 1 (5.3) |
| Cerebral contusion, small | 4 (2.1) | 2 (10.5) |
| Cerebral contusion, tiny | 2 (1.0) | 1 (5.3) |
| Concussion | 76 (39.0) | 1 (5.3) |
| Epidural hematoma, large | 2 (1.0) | 0 (0.0) |
| Epidural hematoma, small or moderate | 4 (2.1) | 0 (0.0) |
| Intracerebral hematoma, large | 1 (0.5) | 0 (0.0) |
| Scalp abrasion | 1 (0.5) | 0 (0.0) |
| Scalp laceration | 4 (2.1) | 0 (0.0) |
| Subarachnoid hemorrhage w/o coma | 9 (4.6) | 1 (5.3) |
| Subdural hematoma, tiny | 27 (13.8) | 3 (15.8) |
| Subdural hematoma, large/extensive | 10 (5.1) | 2 (10.5) |
| Subdural hematoma, small/moderate | 18 (9.2) | 5 (26.3) |
| Subdural hematoma, tiny | 24 (12.3) | 1 (5.3) |
| Traumatic pneumocephalus | 2 (1.0) | 0 (0.0) |
| Vault fracture | 7 (3.6) | 1 (5.3) |
